# Immunoregulatory Role of NK Cells in Tissue Inflammation and Regeneration

**DOI:** 10.3389/fimmu.2017.00301

**Published:** 2017-03-20

**Authors:** Annie Tosello-Trampont, Fionna A. Surette, Sarah E. Ewald, Young S. Hahn

**Affiliations:** ^1^Beirne B. Carter Center for Immunology Research, Charlottesville, VA, USA; ^2^Department of Microbiology, University of Virginia, Charlottesville, VA, USA

**Keywords:** liver, NK cells, fibrosis, immunoregulation, regeneration

## Abstract

NK cells represent an important first line of defense against viral infection and cancer and are also involved in tissue homeostasis. Studies of NK cell activation in the last decade have revealed that they are able to respond to the inflammatory stimuli evoked by tissue damage and contribute to both progression and resolution of diseases. Exacerbation of the inflammatory response through interactions between immune effector cells facilitates the progression of non-alcoholic fatty liver disease (NAFLD) into steatosis, cirrhosis, and hepatocellular carcinoma (HCC). When hepatic damage is incurred, macrophage activation is crucial for initiating cross talk with neighboring cells present in the liver, including hepatocytes and NK cells, and the importance of this interaction in shaping the immune response in liver disease is increasingly recognized. Inflicted structural damage can be in part regenerated *via* the process of self-limiting fibrosis, though persistent hepatic damage will lead to chronic fibrosis and loss of tissue organization and function. The cytotoxic activity of NK cells plays an important role in inducing hepatic stellate cell apoptosis and thus curtailing the progression of fibrosis. Alternatively, in some diseases, such as HCC, NK cells may become dysregulated, promoting an immunosuppressive state where tumors are able to escape immune surveillance. This review describes the current understanding of the contributions of NK cells to tissue inflammation and metabolic liver diseases and the ongoing effort to develop therapeutics that target the immunoregulatory function of NK cells.

## Introduction

The liver plays a critical role in functioning as both a metabolic and immunological site. It receives a dual blood supply; one-quarter is oxygen-rich blood delivered by the hepatic artery, while the remaining three-quarters is blood draining from the gastrointestinal tract and spleen *via* the portal vein, enriched in dietary- and environmental-antigen (1). Liver sinusoidal endothelial cells (LSECs) form the walls of hepatic sinusoids and present numerous fenestrations, allowing blood to contact the underlying hepatocytes. Slow blood flow in hepatic sinusoids allows a better interaction between circulating lymphocytes, liver sinusoidal endothelium, and hepatocytes to facilitate the clearance of gut-derived antigens by liver-resident cells (2). To compensate for the high exposure to circulating antigens, the liver must maintain a tolerant microenvironment in which there is constant low-level suppression of immune responses. Liver immune cells are educated to permit immunological tolerance to self-antigens, environmental, and dietary antigens, during homeostasis, but can initiate both innate and adaptive immune responses in the context of infection (3). In humans and mice, the liver is largely composed of hepatocytes (80% of the liver mass), while the remaining 20% is made up of non-parenchymal cells including lymphocytes, myeloid cells, Kupffer cells (liver-resident macrophages, KCs), HSCs, and LSECs (4, 5). NK cells are enriched in the liver, representing 25–30% of human liver lymphocytes compared to 10–20% of total peripheral blood mononuclear cell (PBMC) lymphocytes (6). However, during chronic hepatitis B and C, NK cell numbers are increased through recruitment by KC-secreted chemokines (7, 8), and the survival of NK cells is enhanced by cytokine production from Kupffer cells, LSECs, and T cells (9). The high immunological load present during infection, a large proportion of which are NK cells, results in a unique immune environment.

NK cells are widely distributed in both lymphoid (bone marrow and liver) and non-lymphoid organs (peripheral blood, lung, and uterus) and bridge the gap between innate and adaptive immune responses. They conduct immunosurveillance by probing cells *via* their inhibitory receptors [NKG2A and the Ly-49 family in mice, and killer-immunoglobulin-like receptor (KIR) and NKG2A in humans] to determine whether the correct self major histocompatibility complex (MHC) is expressed and to ensure tolerance against healthy cells. In humans and mice, NK cells can detect infected, transformed, or stressed cells with their activating receptors (NKG2D and NKp46), resulting in their activation. NK cell activation can be triggered many ways, including cross-linking of activating receptors (NKG2D and NKp46) with simultaneous disengagement of inhibitory receptors (NKG2A) or by various cytokines such as type I IFNs, IL-2, IL-12, IL-15, and IL-18. Additionally, NK cells can be directly activated through CD16A signaling that triggers antibody-dependent cell-mediated cytotoxicity (ADCC) or receive signals through toll-like receptors (TLRs) expressed on their surface, which recognize pathogen-associated molecular patterns (PAMPs) expressed by injured cells (10). Upon activation, NK cells can become cytotoxic and release lytic granules (perforin, granzymes) or induce death signals through expression of death receptors (TRAIL/TRAIL-R, FasL/Fas) (11, 12). While NK cells are able to mediate their functions in an antigen-independent, innate manner, recent investigations have suggested that liver-resident NK cells are capable of acquiring antigen-specific memory. In studies that utilized murine models, it was shown that a persistent and transferable NK cell memory response is generated to haptens and viruses and that the retention of this memory population requires CXCR6 expression (13). This antigen-specific NK memory has further been studied in non-human primates, where it has been maintained up to 5 years (14). However, the underlying mechanisms for the generation of NK memory responses still remain to be elucidated.

**Figure 1:**
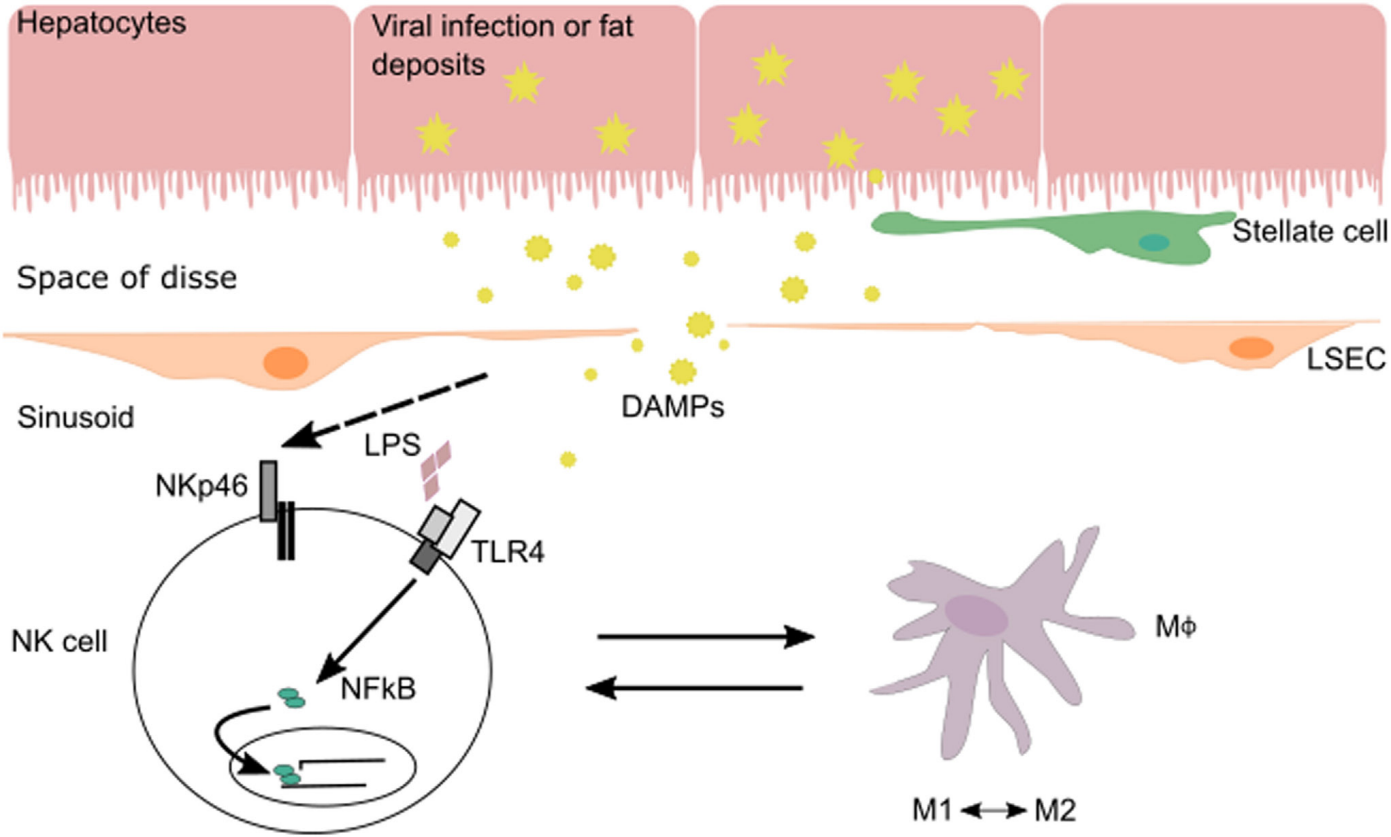
**In the context of viral infection or fatty liver disease, NK cells can be stimulated *via* their activating receptors or by toll-like receptors (TLRs) that recognize pathogen-associated molecular patterns and DAMPs expressed by injured cells**. During liver injury-induced inflammation, NK cells are able to regulate inflammatory (M1) and anti-inflammatory (M2) macrophages depending on the phase of the immune response and stage of disease.

The interplay between NK cells and their surrounding tissues and immune cells shapes NK cell maturation and function. In the liver, cross talk between NK cells and macrophages during various phases of liver injury-induced inflammation allows NK cells to regulate both inflammatory and anti-inflammatory macrophages (Figure 1). Hepatic macrophages play a central role in the pathogenesis of chronic liver disease (15). They can exert dual responses depending on their origin, the phase of liver immune response, the developmental stage of a disease, or the acute/chronic profile of a disease. Macrophages can differentiate into a wide range of pro-inflammatory/classical M1 to immunoregulatory/alternative M2 macrophage profile under inflammatory conditions during liver injury (16). M1 macrophages are activated by pro-inflammatory cytokines (IFN-γ, IL-12, or TNF-α) and/or microbial products (LPS) and can phagocytose bacteria and virus, as well as release pro-inflammatory cytokines and chemokines. M2 macrophages are induced by IL-4, IL-10, and IL-13 produced by various cell types (16). There are several subtypes of M2 macrophages whose functions vary between wound healing (fibrogenic activity and tissue repair), restorative function (matrix resolution and tissue repair), or pro-inflammatory (“turnoff immune response”) activity.

Moreover, NK cell function can be shaped by inflammasomes, which are triggered in response to cell damage. Inflammasome components are expressed in hepatocytes, KCs, and macrophages and contribute to the induction of liver inflammation *via* pyroptotic cell death and release of alarmins as well as the cleavage and secretion of IL-1β and IL-18. The inflammasome sensor NLRP3 responds to extracellular ATP released from dying cells and to reactive oxygen species triggered by phagosome damage from engulfment of particulates (17, 18). Mice deficient in NLRP3 are protected in diet-induced and some infection models of liver injury (19–24). Viral double-stranded DNA can trigger the inflammasome by binding AIM2 expressed on hepatocytes (25). In the case of hepatitis C virus (HCV) infection, both hepatocytes and KC have been shown to release IL-1β and IL-18. Notably, KCs appear to be responsible for activating NK cells *via* this mechanism (25–27). While cell death and release of alarmins exacerbate liver inflammation, IL-18 exerts a protective function to limit liver injury. This may in part be due to the NK cell-mediated activation of other immune mediators, including the induction of PD-L1 and FasL, as well as the essential role of cytokine in eliciting NK memory (28–31). These findings support the pathophysiological role of inflammasomes in hepatic inflammation and liver injury.

## Characteristics of Liver NK Cells

Liver NK cells are a unique cell population in terms of frequency, phenotype, and function (32). Due to its anatomical structure, blood supply, immune resident cell repertoire, and the constant exposure to dietary- and gut-derived antigens, the tolerogenic liver microenvironment may be conducive for the enrichment and characteristics of liver NK cells (33). While the majority of NK cells originate in bone marrow from progenitor NK cells (NKPs), some NK cells evolve in other lymphoid tissues such as the thymus and lymph nodes (34, 35). Compared to bone marrow-derived NK cells, NK cells developing from the thymus demonstrate a higher expression of GATA-3 and CD127 and require IL-7 signals for their development (34). The earliest committed NKPs in mouse bone marrow are identified as DX5^−^CD161^−^CD122^+^ and are dependent on the Id2 transcription factor (36). NKPs then acquire the expression of phenotypic markers NK1.1, NKp46, CD94/NKG2 receptors, Ly49 receptors, and DX5 to become mature NK cells. The expression of CD11b and CD27 are used to define the maturation status of mouse NK cells. NK cell maturation is categorized into four stages based on the acquisition of CD11b expression and the loss of CD27. The most immature NK cells are defined as CD11b^−^CD27^−^, and these progress to CD11b^−^CD27^+^, then CD11b^+^CD27^+^, and finally become the most mature NK cells by expressing CD11b^+^CD27^−^. Mature CD11b^+^ CD27^−^NK cells are predominant in the spleen, and immature CD11b^−^CD27^+^ NK cells are more prevalent in the liver (37).

The liver is populated by conventional NK (cNK) cells and resident NK cells that are distinguished by the mutually exclusive expression of the integrins DX5 and CD49a, respectively. Mouse NK cells are usually identified by the cell surface phenotype of CD3^−^DX5^+^. The expression of NK activating receptor, NKp46, is also used as a phenotypic marker in mice and humans, with cNK cells defined by the phenotype of CD49a^−^NKp46^+^DX5^+^CD3^−^, and liver-resident NK cells identified by CD49a^+^NKp46^+^DX5^−^CD3^−^, with low expression of CD11b and Ly49, and high expression of TRAIL (38). During homeostasis, cNK cells are highly migratory, circulating throughout the body and primarily found in bone marrow, blood, and spleen. Activated cNK cells produce a large amount of cytokines but display low cytolytic activity. Recently, liver-resident NK cells have been identified as members of innate lymphoid cells (ILCs) and constitute the group 1 ILCs, along with ILC1s (39–42). Both NK cells and ILC1s are characterized by their expression of the transcription factor T-bet, but NK cells are distinctive in their expression of EOMES. While not in the scope of this review, there are numerous others that cover ILC biology, nomenclature, and the vast differences in transcriptional regulation, receptor expression, and localization that may be helpful (43–45). Liver-resident NK cells have more cytotoxicity, but less IFN-γ production following IL-12/IL-18 stimulation than NK cells in the blood and spleen under homeostatic conditions (31). In contrast, a recent report demonstrates that ILC1s are able to produce higher levels of cytokines (IFN-γ) than cNK cells following PMA/ionomycin stimulation (46). Within the liver, CD49a^+^DX5^−^ NK cells are found at a significantly higher frequency compared to other sites (bone marrow, spleen, blood) and selectively reside in hepatic sinusoids. Liver CD49a^+^DX5^−^ NK cells express high levels of TRAIL and have cytotoxic activity against tumor cells. TRAIL^+^ NK cells predominate in fetal and neonatal mice, and in adulthood are present in the liver, but not the spleen. Tissue-resident CD49a^+^DX5^−^ NK cells are also found in the uterus and skin (46).

**Figure 2:**
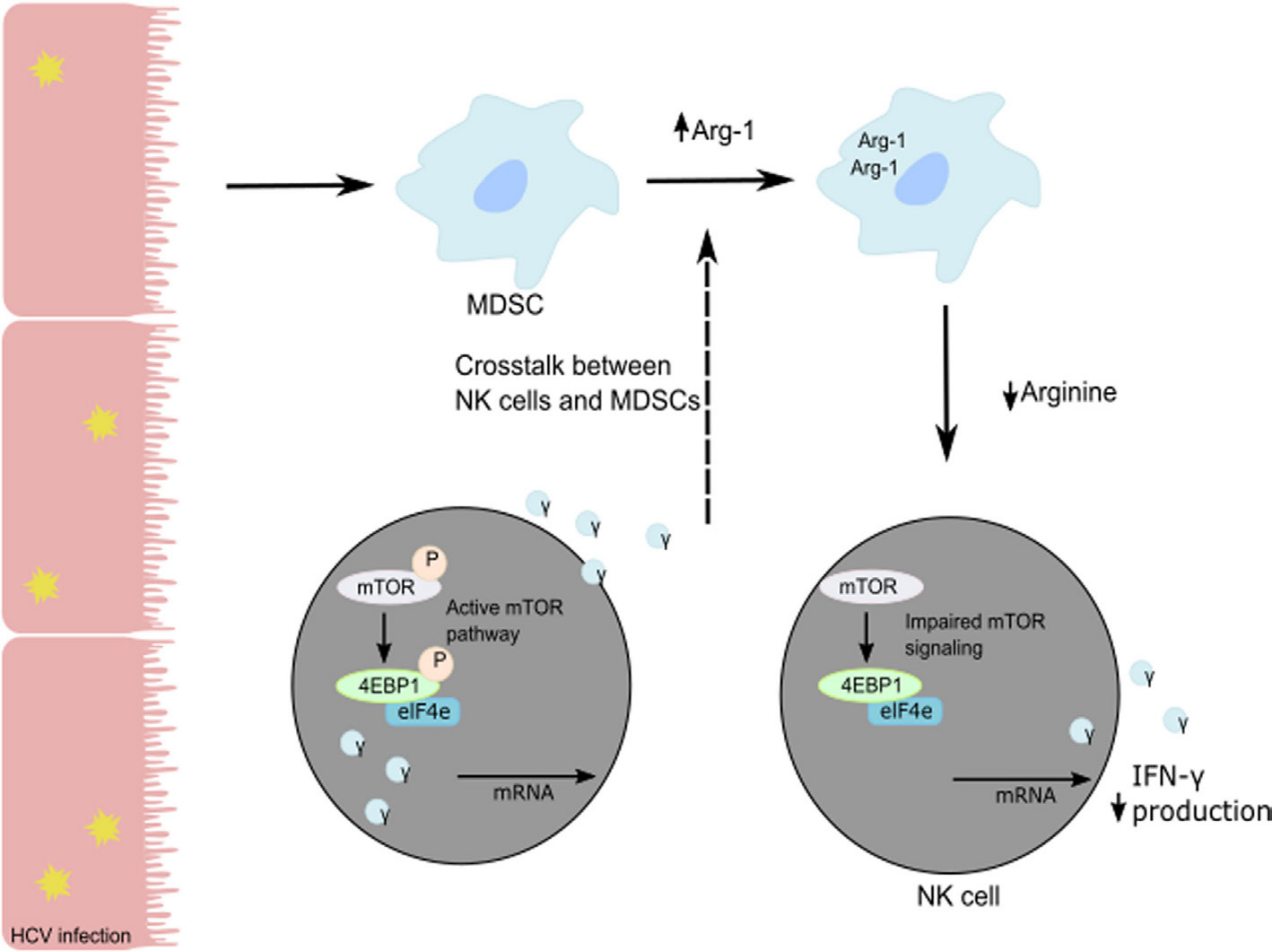
**Suppression of NK cell-derived IFN-γ production is mediated by CD33^+^CD11b^lo^HLA-DR^lo^ myeloid-derived suppressor cells (MDSCs) following a reduction in available arginine *via* an Arg-1 mechanism**. During HCV infection, HCV core protein induces MDSC development. Following the loss of arginine, the mTOR pathway is unable to efficiently translate IFN-γ mRNA, and the subsequent loss of IFN-γ protein production and secretion from NK cells in the liver results in immune evasion by HCV.

Human NK cells are CD3^−^CD56^+^ lymphocytes and can be divided into two subsets based on the level of expression of CD56 and CD16. CD16^+^CD56^dim^ NK cells represent 90% of blood and spleen NK cells and demonstrate higher cytotoxic activity than CD16^−^CD56^bright^ NK cells by producing high levels of granzymes and perforin. CD16^+^CD56^dim^ NK cells have high expression of KIR and express intermediate-affinity IL-2 receptor resulting in low expansion capacity under IL-2 stimulation. Representing only 10% of blood NK cells, CD16^−^CD56^bright^ NK cells are predominant in secondary lymphoid organs such as lymph nodes. In the liver, both populations are present with the same frequency. CD16^−^CD56^bright^ NK cells exhibit less cytolytic activity than CD16^+^CD56^dim^ NK cells but demonstrate the same cytotoxicity after prolonged activation. CD16^−^CD56^bright^ NK cells express high- and intermediate-affinity IL2-receptor facilitating their *in vivo* and *in vitro* expansion under low doses of IL-2. Additionally, they are more responsive to stimulation by pro-inflammatory cytokines. Upon activation, they produce cytokines (IFN-γ, IL-10, GM-CSF, and TNF-α) and chemokines. A recent study suggested that CD16^−^CD56^bright^ NK cells might represent liver-resident NK cells with high expression of CD69, an activation marker, and expression of CXCR6 and CCR5 to retain them in the liver (47). The development and the differentiation of human liver-resident NK cells are still undefined. As an antiviral effect, NK cells secrete granzyme B and perforin to lyse virally infected cells and induce their apoptosis and secrete cytokines such as TNF-α and IFN-γ to further the immune response (48). In particular, NK IFN-γ production has direct cytotoxic effects on virally-infected cells, elicits an antiviral state in the uninfected cells, and induces chemotaxis to recruit adaptive immune cells (6, 49, 50). HCV patients with chronic infection show poor NK cell responses compared to patients with resolved HCV infection. The *in vitro* studies on co-cultures of NK cells with HCV-conditioned CD33^+^ PBMCs demonstrated a reduced level of IFN-γ production but no effect on granzyme B release. This suppression of NK cell-derived IFN-γ production has been shown to be mediated by CD33^+^CD11b^lo^HLA-DR^lo^ myeloid-derived suppressor cells (MDSCs) *via* an arginase-1-dependent inhibition of mTOR activation (Figure 2) (51). These results identify the induction of MDSCs in HCV infection as a potent immune evasion strategy that suppresses antiviral NK cell responses.

## Role of NK Cells in Liver Disease

As a result of tight regulatory controls, loss of liver tissue can be regenerated *via* the process of self-limiting fibrosis (3). Indeed, the liver can be fully recovered structurally and functionally even after a loss of 50% of its hepatocytes. While fibrosis can be beneficial in an acute sense, repeated hepatic damages due to persistent inflammation or infection can lead to chronic fibrosis, resulting in a loss of liver tissue organization and function and eventually, cirrhosis (3). At this stage, if cirrhosis fails to regress to early stage fibrosis, there is a high risk for developing irreversible hepatocellular carcinoma (HCC). The most common causes of fibrosis are chronic viral infections (HBV, HCV), frequent use of hepatotoxic drugs (acetaminophen, ibuprofen), or metabolic syndromes [non-alcoholic fatty liver disease (NAFLD)] that trigger chronic inflammation. Fibrosis results in activation of HSCs and macrophages that produce extracellular matrix (ECM), forming scar tissue. As a result of liver damage, dying hepatocytes are cleared by liver macrophages, which trigger an inflammatory response by secreting cytokines and chemokines to recruit other cells to the wound site and initiate the process of tissue repair. In an inflamed hepatic microenvironment, HSCs become activated and differentiate into myofibroblasts that produce a large amount of fibrous proteins (collagen, elastin, laminin, fibronectin). The progression of fibrosis depends on the balance between production of ECM components and their degradation by metalloproteinase produced by restorative M2 macrophages. In this review, we will focus on the dual role of NK cells in the initiation, progression, and resolution of liver fibrosis and how it is regulated by the cross talk of NK cells with surrounding macrophages and stellate cells.

### Role of NK Cells in Fibrosis

**Figure 3:**
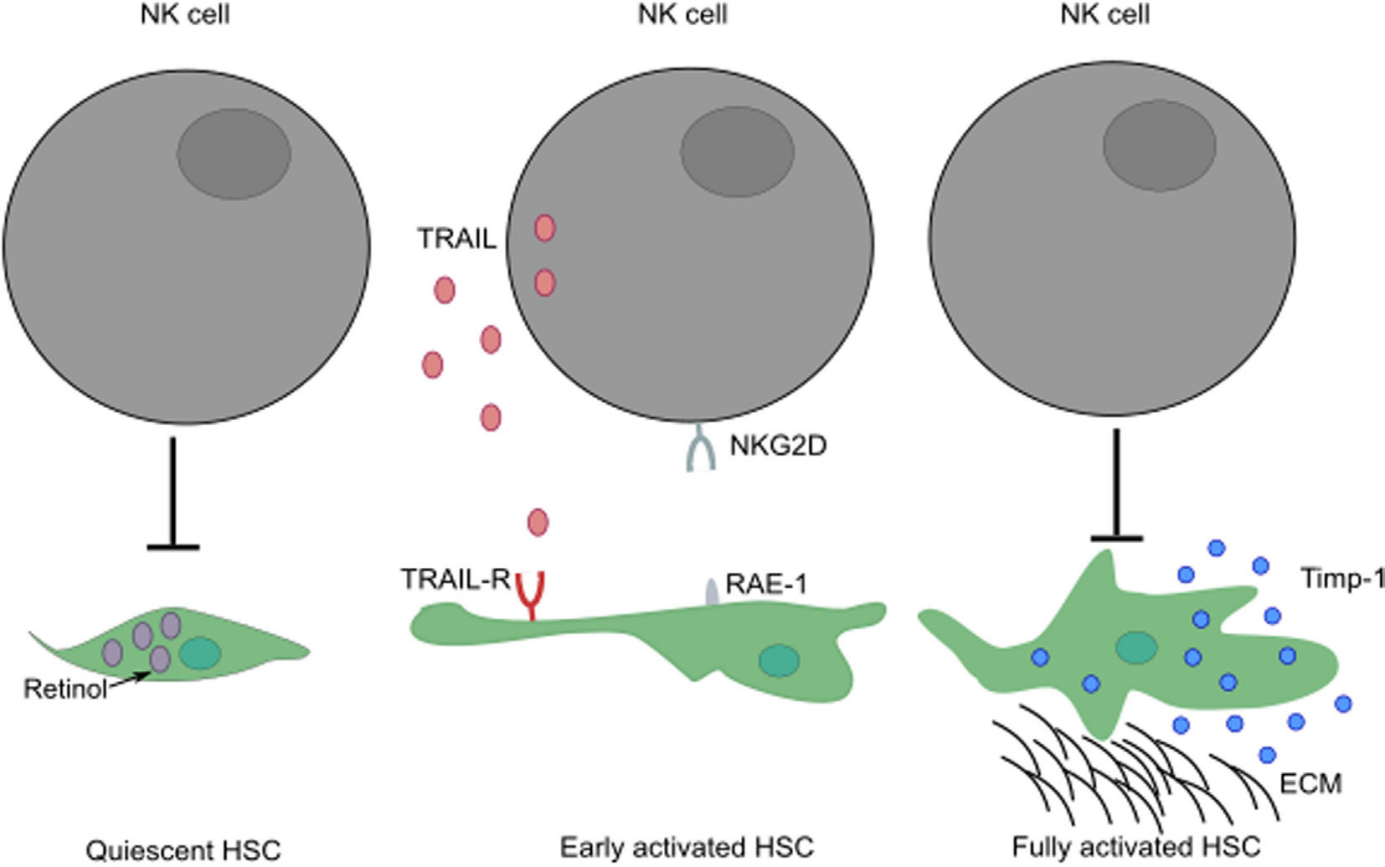
**The activation stage of HSCs can influence the ability of NK cells to exert cytotoxic activity**. While quiescent HSCs are resistant to apoptotic signals, their activation leads to increased surface expression of TRAIL-R. Following engagement of their activating receptors, NK cells can conduct cytotoxic activity by releasing lytic granules or by transduction of death signals through death receptor/ligand interactions (i.e., TRAIL/TRAIL-R, FasL/Fas). Fully activated HSCs expressing Timp-1 are protected from apoptosis. These HSCs can differentiate into myofibroblasts that produce large amounts of fibrous proteins, which can lead to fibrosis when not restrained. ECM, extracellular matrix.

NK cells play a paradoxical role in the development of liver fibrosis. The cytotoxic activity of NK cells can curtail the development of fibrosis by killing HSC-derived myofibroblasts through engagement of NKG2D receptor with its ligand, RAE-1 (in mouse) expressed by early activated HSCs (52, 53). In humans, NK cells preferentially kill senescent-activated HSCs expressing MICA following recognition by NKG2D receptor on NK cells (54, 55). In addition, the expression of NKp46 ligand on human and mouse-activated HSCs can also trigger NK cell-mediated cytotoxic activity, ameliorating liver fibrosis (56). The ability of NK cells to kill HSCs seems to be dependent on the activation stage of HSCs and expression of specific molecules; quiescent HSCs and activated HSCs that express Timp-1 are resistant to apoptotic signals (57) (Figure 3). HSCs are the most sensitive to NK cytotoxicity during early activation and senescence stages.

In humans and mice, IFN-γ-producing NK cells have been demonstrated to negatively regulate fibrosis. *In vitro* and *in vivo* studies in mouse have shown an anti-fibrotic effect of NK cell-derived IFN-γ, which induces HSC apoptosis and cell cycle arrest. However, clinical trials with IFN-γ proved ineffective; the anti-fibrotic action of IFN-γ might depend on its targeted delivery to HSCs (58, 59). Additionally, IFN-γ-producing DX5^+^NKp46^+^ cNK cells are increased during non-alcoholic steatohepatitis (NASH), a cause of fibrosis, and skew the polarization of activated KC and liver macrophages toward an M1 profile, rather than toward a fibrosis-inducing M2 macrophage profile (60). Furthermore, NK cells release IL-22 upon activation, which can be anti-fibrogenic (61–63).

While IL-22 plays a beneficial role during acute inflammatory events, prolonged and excessive production of IL-22 may have the opposite effect (62). Continuous exposure to proliferative and antiapoptotic signals may drive cells to change phenotype and become cancerous as demonstrated in IL-22 transgenic mice where IL-22 primes the liver to be more susceptible to tumor development (64). Additionally, in HBV-infected patients and HBV transgenic mice, IL-22 exacerbates chronic inflammation and the development of fibrosis by promoting Th17 cell responses in the liver (65–68). The dual nature of IL-22, and by extension, NK cells, between protection and inflammation may depend on the tissue and inflammatory conditions. Due to technical challenges, it is difficult to evaluate which specific NK cell subset (i.e., conventional or liver-resident NK cells) has anti-fibrogenic effects. Most of the *in vitro* studies use cNK cells from human blood or mouse spleens and *in vivo* depletion experiments using antibodies do not efficiently remove all liver-resident NK cells. Few liver-resident NK cells can be purified from a mouse liver, significantly prohibiting adoptive transfer.

In contrast to the anti-fibrogenic properties of some NK cells, other studies have demonstrated that NK cells can enhance liver injury by killing stressed hepatocytes *via* engagement of NKG2D, NKp30, and/or TRAIL. Additionally, they can accomplish this *via* IFN-γ-induced apoptosis, leading to the development of fibrosis (69–74). For example, patients with NASH show an increase in hepatocyte Fas expression and apoptosis, which correlates with the severity of the disease (75). This pathogenic role of NK cells has also been identified in human autoimmune liver diseases, such as primary biliary cirrhosis, where NK cells promote the killing of biliary epithelial cells *via* TRAIL and by releasing cytokines that promote adaptive immunity (76, 77). In parallel, IL-10-producing NK cells delay primary biliary cirrhosis by annihilating the adaptive immune response in killing autologous DCs and T cells (78).

### Role of NK Cells in Tissue Regeneration

As many studies have now demonstrated the regulatory role of NK cells in fibrosis, compelling evidence also points to a role for NK cells in tissue regeneration (79, 80). Hepatic NK cells can interact with surrounding parenchymal and non-parenchymal cells to influence the release of growth hormones, cytokines, and chemokines within the proliferating hepatic tissues. Hepatic NK cells promoted liver regeneration after partial hepatectomy (81). NK cells may have a beneficial effect on liver tissue regeneration by secreting TNF-α and IL-22 during inflammation, which stimulate the proliferation of hepatocytes to replace dying hepatocytes (64, 82). NK cells might also play a role in liver regeneration by cross talk with hepatic macrophages known to regulate the differentiation of hepatic progenitor cells into hepatocytes (83). All of the regenerative effects of NK cells are dependent on optimal NK cell activation, as overactivation actually prevents liver regeneration (84). Indeed, when strongly activated, NK cells produce excessive amounts of IFN-γ and lose self-tolerance, compromising liver repair (85). To maintain self-tolerance during regenerative hyperplasia, hepatocytes upregulate the poliovirus receptor (PVR/CD155) after partial hepatectomy, which engages the regulatory molecule T cell Ig and ITIM domain (TIGIT) on NK cells (84). NK cell effector functions used to resolve inflammation also shape the recruitment and differentiation of stem cells for tissue regeneration. NK cells enhance the recruitment of mesenchymal stem cells (MSCs) by producing a variety of chemokines, such as, NAP-2 (CXCL7), GRO-b (CXCL2), GRO-g (CXCL3), IL-8, and RANTES (86). MSCs have the capacity to differentiate into specific lineages that promote tissue repair. The cross talk between NK cells and MSCs has been demonstrated in several studies, in particular, during bone repair where NK cells mediate the recruitment of MSCs. Taken together, the dual role of NK cells on fibrosis and tissue regeneration could be explained by their differentiation from actively cytotoxic cells to regulatory cells that produce cytokines supportive of tissue repair. Regulatory NK cells might be induced as a result of NK cell interactions with surrounding cells such as immune cells (KC and liver macrophages), effector cells of connective tissue (fibroblasts, HSCs), or stem cells.

### Role of NK Cells in HCC

In mouse models of cancer and human cancer patients, NK cells are often dysfunctional, with reduced cytotoxic activity, impaired production of cytokines, and an inability to efficiently kill abnormal cells (87–90). In patients with HCC, there is also a significant reduction in both the percentage and number of total liver NK cells, as well as peripheral CD56^dim^CD16^pos^ subsets (91, 92). At all stages of HCC, these CD56^dim^CD16^pos^ NK cells have a decreased ability to produce cytokine, with IFN-γ production following PMA/ionomycin stimulation decreasing from 50 to 5% between healthy and HCC donors (93). Total NK cells show reduced production of Granzyme A, Granzyme B, and perforin once HCC has progressed past stage I (93). NK cells cultured with cancer-associated fibroblasts from HCC (H-CAFs) downregulate NKG2D and NKp46 and decrease expression of Granzyme B, perforin, TNF-α, and INF-γ (94). Following PGE2 blockade by the inhibitor NS398, these NK cells increased TNF-α and IFN-γ production, an effect that was further amplified with the addition of the indoleamine 2,3-dioxygenase (IDO) inhibitor, 1-MT (94). Further investigation into the interactions that contribute to dysregulated NK cells and by extension, the immunosuppressive state that facilitates HCC progression, is necessary to fully understand the tumor microenvironment and how to target therapies to these interactions.

## Therapeutics Targeted to NK Cells

The immunoregulatory function of NK cells in infection and inflammatory diseases makes these cells good therapeutic targets for controlling infection and preventing the development of chronic inflammatory diseases (88). Several preclinical therapeutic approaches have been used to target NK cells for treating HCC. Therapies included induction of the activation of NK cells (with cytokines such as IL-2, IL-12, IL-15, IL-21, IFN-α, and IFN-γ) or performing adoptive transfers of activated NK cells. Type I IFNs has been shown to have antiviral, anti-fibrotic, and antitumor effects, likely due to their ability to stimulate NK cytolytic activity (37). IL-12 and IL-18 are also potent activators of NK cells that produce IFN-γ and TNF-α and have antiviral effects (95). Activation can also be achieved by blocking NK cell inhibitory receptors with monoclonal antibodies (mAb) (anti-NKG2A or anti-KIR), or by activating NK activating receptors to enhance NK cell ADCC against tumor cells.

Therapeutic approaches have been designed to use specific mAb to target co-inhibitory molecules (i.e., immune checkpoint inhibitors) that stop immune responses against tumor cells (96). Blocking these immune checkpoint inhibitors would release immune cells, including NK cells, from inhibitory mechanisms to restore their full immune activity. These immune checkpoint inhibitors are well described in the context of T cell exhaustion (97, 98). They include cytotoxic T-lymphocyte antigen 4 (CTLA-4), programmed death 1 (PD-1), lymphocyte activation gene-3 (LAG-3), T-cell immunoglobulin and mucin-domain-containing-3 (TIM-3), and B and T lymphocyte attenuator (BTLA). The expression of PD-1 and Tim-3 on liver NK cells from patients with HBV-related HCC is increased and also associated with immune cell exhaustion (99, 100). The promising results from clinical trials using mAb against immune checkpoint inhibitors might also prove to be a likely therapeutic strategy for liver disease, especially in combination with drugs enhancing NK cell activation. Recent studies have shown that the expression of Tim-3 on peripheral CD56^+^ NK cells correlates with expression of serological markers of liver fibrosis in patients with advanced schistosomiasis (101). Blocking PD-1, CTLA-4, or Tim-3 pathway with mAbs could protect patients from fibrosis, cirrhosis, and HCC by restoring NK cell function. Several mAbs against PD-1 (ipilimumab, pembrolizumab, nivolumab), PD-L1 (atezolizumab, durvalumab), or CTLA-4 (tremelimumab/CP-675,206) are currently being tested in clinical trials against HCC.

NK cell activation can also be achieved through blocking NK inhibitory receptors such as KIR, NKG2A/CD94, or TIGIT. Cancer cells or virus-infected cells upregulate the expression of MHC class I molecules (HLA in humans) as an immune escape mechanism in order to evade killing by NK and T cells. MHC I molecules in mice, and HLA molecules in humans, engage NK inhibitory receptors like Ly49 and NKG2A and KIR, respectively, to prevent NK cell activation. Anti-NKG2A antibodies are already under investigation in several clinical trials alone, or in combination with other anticancer drugs (ibrutninb), mAb-like anti-EGFR (cetuximab), or anti-PD-L1 (duvalumab). New clinical studies are in progress to test the antitumor effect of anti-KIR monoclonal antibody in combination with other drugs.

Taken together, the combination of NK cell-based therapy with conventional therapies might become an efficient approach to cure or lessen the burden of liver cancers. More clinical trials are needed to evaluate the safety and efficiency of these combined approaches. In addition, NK cells play different roles depending on the developmental stage of a specific disease, emphasizing the importance of investigating the spatiotemporal role of NK cells in cancer and fibrosis. It is worth noting that most NK cell studies are done with peripheral and splenic NK cells, not with tissue-resident NK cells. More investigations are required to determine the specific role of each NK cell subset, how these subsets contribute to the development of tissue-specific disease, and the most effective therapeutic strategies for each disease.

## Summary and Conclusion

NK cells play crucial roles in regulation of chronic inflammatory diseases such as tissue fibrosis and cancer. Thus, the understanding of NK-mediated immunoregulation would provide insight into designing therapeutics against viral infection and inflammatory diseases. NK cells have a protective role in the development of liver fibrosis in the model of NAFLD development where they regulate the tight balance between liver inflammation and repair through macrophage polarization. Thus, the identification of NK cells as upstream regulators of macrophage function provides a new cellular target to modulate macrophage-mediated inflammation in chronic liver diseases. Further exploration of the interplay between myeloid cells and NK cells may thus help identify key molecular regulators that can resolve chronic inflammation and restore immune homeostasis.

## Author Contributions

AT-T reviewed the literature and wrote the manuscript. YH, FS, and SE contributed to and edited the manuscript.

## Conflict of Interest Statement

The authors declare that the research was conducted in the absence of any commercial or financial relationships that could be construed as a potential conflict of interest.

## References

[1] Jenne CN Kubes P. Immune surveillance by the liver. Nat Immunol (2013) 14(10):996–1006. 10.1038/ni.2691 24048121

[2] Racanelli V Rehermann B. The liver as an immunological organ. Hepatology (2006) 43(2 Suppl 1):S54–62. 10.1002/hep.21060 16447271

[3] Pellicoro A Ramachandran P Iredale JP Fallowfield JA. Liver fibrosis and repair: immune regulation of wound healing in a solid organ. Nat Rev Immunol (2014) 14(3):181–94. 10.1038/nri3623 24566915

[4] Kmieć Z. Cooperation of liver cells in health and disease. Adv Anat Embryol Cell Biol (2001) 161:1–151. 10.1007/978-3-642-56553-3 11729749

[5] Mehal WZ Azzaroli F Crispe IN Immunology of the healthy liver: old questions and new insights. Gastroenterology (2001) 120(1):250–60. 10.1053/gast.2001.20947 11208734

[6] Żeromski J Mozer-Lisewska I Kaczmarek M Kowala-Piaskowska A Sikora J. NK cells prevalence, subsets and function in viral hepatitis C. Arch Immunol Ther Exp (2011) 59(6):449–55. 10.1007/s00005-011-0145-y 21972016

[7] Bonorino P Ramzan M Camous X Dufeu-Duchesne T Thelu MA Sturm N Fine characterization of intrahepatic NK cells expressing natural killer receptors in chronic hepatitis B and C. J Hepatol (2009) 51(3):458–67. 10.1016/j.jhep.2009.05.030 19596474

[8] Oliviero B Varchetta S Paudice E Michelone G Zaramella M Mavilio D Natural killer cell functional dichotomy in chronic hepatitis B and chronic hepatitis C virus infections. Gastroenterology (2009) 137(3):1151–60. 10.1053/j.gastro.2009.05.047 19470388

[9] Gregoire C Chasson L Luci C Tomasello E Geissmann F Vivier E The trafficking of natural killer cells. Immunol Rev (2007) 220:169–82. 10.1111/j.1600-065X.2007.00563.x 17979846 PMC7165697

[10] Kawai T Akira S. The role of pattern-recognition receptors in innate immunity: update on toll-like receptors. Nat Immunol (2010) 11(5):373–84. 10.1038/ni.1863 20404851

[11] Hamerman JA Ogasawara K Lanier LL NK cells in innate immunity. Curr Opin Immunol (2005) 17(1):29–35. 10.1016/j.coi.2004.11.001 15653307

[12] Takeda K Hayakawa Y Smyth MJ Kayagaki N Yamaguchi N Kakuta S Involvement of tumor necrosis factor-related apoptosis-inducing ligand in surveillance of tumor metastasis by liver natural killer cells. Nat Med (2001) 7(1):94–100. 10.1038/83416 11135622

[13] Paust S Gill HS Wang B Flynn MP Moseman EA Senman B Critical role for the chemokine receptor CXCR6 in NK cell-mediated antigen-specific memory of haptens and viruses. Nat Immunol (2010) 11(12):1127–35. 10.1038/ni.1953 20972432 PMC2982944

[14] Reeves RK Li H Jost S Blass E Li H Schafer JL Antigen-specific NK cell memory in rhesus macaques. Nat Immunol (2015) 16(9):927–32. 10.1038/ni.3227 26193080 PMC4545390

[15] Schuppan D Kim YO. Evolving therapies for liver fibrosis. J Clin Invest (2013) 123(5):1887–901. 10.1172/JCI66028 23635787 PMC3635731

[16] Labonte AC Tosello-Trampont AC Hahn YS The role of macrophage polarization in infectious and inflammatory diseases. Mol Cells (2014) 37(4):275–85. 10.14348/molcells.2014.2374 24625576 PMC4012075

[17] Duewell P Kono H Rayner KJ Sirois CM Vladimer G Bauernfeind FG NLRP3 inflammasomes are required for atherogenesis and activated by cholesterol crystals. Nature (2010) 464(7293):1357–61. 10.1038/nature08938 20428172 PMC2946640

[18] He Y Hara H Nunez G Mechanism and regulation of NLRP3 inflammasome activation. Trends Biochem Sci (2016) 41(12):1012–21. 10.1016/j.tibs.2016.09.002 27669650 PMC5123939

[19] Guo S Yang C Diao B The NLRP3 inflammasome and IL-1β accelerate immunologically mediated pathology in experimental viral fulminant hepatitis. PLoS Pathog (2015) 11(9):e1005155 10.1371/journal.ppat.1005216 26367131 PMC4569300

[20] Huang H Chen H-W Evankovich J Yan W Rosborough BR Nace GW Histones activate the NLRP3 inflammasome in Kupffer cells during sterile inflammatory liver injury. J Immunol (2013) 191(5):2665–79. 10.4049/jimmunol.1202733 23904166 PMC3777242

[21] Kamo N Ke B Ghaffari A SC/caspase-1/IL-1β signaling triggers inflammatory responses by promoting HMGB1 induction in liver ischemia/reperfusion injury. Hepatology (2013) 58(1):351–62. 10.1002/hep.26320 23408710 PMC3679353

[22] Vandanmagsar B Youm Y-H Ravussin A Galgani JE Stadler K Mynatt RL The NLRP3 inflammasome instigates obesity-induced inflammation and insulin resistance. Nat Med (2011) 17(2):179–88. 10.1038/nm.2279 21217695 PMC3076025

[23] Negash AA Ramos HJ Crochet N Lau DTY Doehle B Papic N IL-1β production through the NLRP3 inflammasome by hepatic macrophages links hepatitis C virus infection with liver inflammation and disease. PLoS Pathog (2013) 9(4):e1003330 10.1371/journal.ppat.1003330 23633957 PMC3635973

[24] Maltez V Tubbs AL Cook KD Aachoui Y Falcone EL Holland SM Inflammasomes coordinate pyroptosis and natural killer cell cytotoxicity to clear infection by a ubiquitous environmental bacterium. Immunity (2015) 43(5):987–97. 10.1016/j.immuni.2015.10.010 26572063 PMC4654968

[25] Zannetti C Roblot G Charrier E. Characterization of the inflammasome in human Kupffer cells in response to synthetic agonists and pathogens. J Immunol (2016) 197(1):356–67. 10.4049/jimmunol.1502301 27226092

[26] Pan X Xu H Zheng C Li M Zou X Cao H Human hepatocytes express absent in melanoma 2 and respond to hepatitis B virus with interleukin-18 expression. Virus Genes (2016) 52(4):445–52. 10.1007/s11262-016-1327-9 27094165

[27] Movita D Van de Garde MD Biesta P Kreefft K Haagmans B Zuniga E Inflammatory monocytes recruited to the liver within 24 hours after virus-induced inflammation resemble Kupffer cells but are functionally distinct. J Virol (2015) 89(9):4809–17. 10.1128/JVI.03733-14 25673700 PMC4403491

[28] Dupaul-Chicoine J Arabzadeh A Dagenais M Douglas T Champagne C Morizot A The Nlrp3 inflammasome suppresses colorectal cancer metastatic growth in the liver by promoting natural killer cell tumoricidal activity. Immunity (2015) 43(4):751–63. 10.1016/j.immuni.2015.08.013 26384545

[29] Terme M Ullrich E Aymeric L Meinhardt K Coudert JD Desbois M Cancer-induced immunosuppression: IL-18-elicited immunoablative NK cells. Cancer Res (2012) 72(11):2757–67. 10.1158/0008-5472.CAN-11-3379 22427351

[30] Terme M Ullrich E Aymeric L Meinhardt K Desbois M Delahaye N IL-18 induces PD-1-dependent immunosuppression in cancer. Cancer Res (2011) 71(16):5393–9. 10.1158/0008-5472.CAN-11-0993 21724589

[31] Van den Boorn JG Jakobs C Hagen C Renn M Luiten RM Melief CJ Inflammasome-dependent induction of adaptive NK cell memory. Immunity (2016) 44(6):1406–21. 10.1016/j.immuni.2016.05.008 27287410

[32] Lassen MG Lukens JR Dolina JS Brown MG Hahn YS Intrahepatic IL-10 maintains NKG2A(+)Ly49(-) liver NK cells in a functionally hyporesponsive state. J Immunol (2010) 184(5):2693–701. 10.4049/jimmunol.0901362 20124099 PMC2885840

[33] Robinson MW Harmon C O’Farrelly C. Liver immunology and its role in inflammation and homeostasis. Cell Mol Immunol (2016) 13(3):267–76. 10.1038/cmi.2016.3 27063467 PMC4856809

[34] Di Santo JP. Natural killer cell developmental pathways: a question of balance. Annu Rev Immunol (2006) 24:257–86. 10.1146/annurev.immunol.24.021605.090700 16551250

[35] Geiger TL Sun JC. Development and maturation of natural killer cells. Curr Opin Immunol (2016) 39:82–9. 10.1016/j.coi.2016.01.007 26845614 PMC4801705

[36] Rosmaraki EE Douagi I Roth C Colucci F Cumano A Di Santo JP. Identification of committed NK cell progenitors in adult murine bone marrow. Eur J Immunol (2001) 31(6):1900–9. 10.1002/1521-4141(200106)31:6<1900::AID-IMMU1900>3.0.CO;2-M 11433387

[37] Krueger PD Lassen MG Qiao HH Hahn YS Regulation of NK cell repertoire and function in the liver. Crit Rev Immunol (2011) 31(1):43–52. 10.1615/CritRevImmunol.v31.i1.40 21395510 PMC3163300

[38] Peng H Jiang X Chen Y Sojka DK Wei H Gao X Liver-resident NK cells confer adaptive immunity in skin-contact inflammation. J Clin Invest (2013) 123(4):1444–56. 10.1172/JCI66381 23524967 PMC3613925

[39] Klose CS Flach M Mohle L Rogell L Hoyler T Ebert K Differentiation of type 1 ILCs from a common progenitor to all helper-like innate lymphoid cell lineages. Cell (2014) 157(2):340–56. 10.1016/j.cell.2014.03.030 24725403

[40] Peng H Tian Z. Re-examining the origin and function of liver-resident NK cells. Trends Immunol (2015) 36(5):293–9. 10.1016/j.it.2015.03.006 25846402

[41] Serafini N Vosshenrich CA Di Santo JP Transcriptional regulation of innate lymphoid cell fate. Nat Rev Immunol (2015) 15(7):415–28. 10.1038/nri3855 26065585

[42] Spits H Bernink JH Lanier L. NK cells and type 1 innate lymphoid cells: partners in host defense. Nat Immunol (2016) 17(7):758–64. 10.1038/ni.3482 27328005

[43] Sonnenberg G Artis D. Innate lymphoid cells in the initiation, regulation and resolution of inflammation. Nat Med (2015) 21(7):698–708. 10.1038/nm.3892 26121198 PMC4869856

[44] Spits H Artis D Colonna M Diefenbach A Di Santo JP Eberl G Innate lymphoid cells – a proposal for uniform nomenclature. Nat Rev Immunol (2013) 13(2):145–9. 10.1038/nri3365 23348417

[45] Jiao Y Huntington ND Belz GT Seillet C. Type 1 innate lymphoid cell biology: lessons learnt from natural killer cells. Front Immunol (2016) 7:426. 10.3389/fimmu.2016.00426 27785129 PMC5059362

[46] Sojka DK Plougastel-Douglas B Yang L Pak-Wittel MA Artyomov MN Ivanova Y Tissue-resident natural killer (NK) cells are cell lineages distinct from thymic and conventional splenic NK cells. Elife (2014) 3:e01659. 10.7554/eLife.01659 24714492 PMC3975579

[47] Hudspeth K Donadon M Cimino M Pontarini E Tentorio P Preti M Human liver-resident CD56(bright)/CD16(neg) NK cells are retained within hepatic sinusoids via the engagement of CCR5 and CXCR6 pathways. J Autoimmun (2016) 66:40–50. 10.1016/j.jaut.2015.08.011 26330348 PMC4718768

[48] Ahlenstiel G. The natural killer cell response to HCV infection. Immune Netw (2013) 13(5):168–76. 10.4110/in.2013.13.5.168 24198741 PMC3817297

[49] Roda JM Parihar R Magro C Nuovo GJ Tridandapani S Carson WE. Natural killer cells produce T cell-recruiting chemokines in response to antibody-coated tumor cells. Cancer Res (2006) 66(1):517–26. 10.1158/0008-5472.CAN-05-2429 16397268

[50] Sancéau J Sondermeyer P Béranger F Falcoff R Vaquero C. Intracellular human gamma-interferon triggers an antiviral state in transformed murine L cells. Proc Natl Acad Sci U S A (1987) 84(9):2906–10. 10.1073/pnas.84.9.2906 3033669 PMC304769

[51] Goh CC Roggerson KM Lee HC Golden-Mason L Rosen HR Hahn YS. Hepatitis C virus-induced myeloid-derived suppressor cells suppress NK cell IFN-γ production by altering cellular metabolism via arginase-1. J Immunol (2016) 196(5):2283–92. 10.4049/jimmunol.1501881 26826241 PMC4761460

[52] Melhem A Muhanna N Bishara A Alvarez CE Ilan Y Bishara T Anti-fibrotic activity of NK cells in experimental liver injury through killing of activated HSC. J Hepatol (2006) 45(1):60–71. 10.1016/j.jhep.2005.12.025 16515819

[53] Radaeva S Sun R Jaruga B Nguyen VT Tian Z Gao B. Natural killer cells ameliorate liver fibrosis by killing activated stellate cells in NKG2D-dependent and tumor necrosis factor-related apoptosis-inducing ligand-dependent manners. Gastroenterology (2006) 130(2):435–52. 10.1053/j.gastro.2005.10.055 16472598

[54] Krizhanovsky V Yon M Dickins RA Hearn S Simon J Miething C Senescence of activated stellate cells limits liver fibrosis. Cell (2008) 134(4):657–67. 10.1016/j.cell.2008.06.049 18724938 PMC3073300

[55] Sagiv A Burton DG Moshayev Z Vadai E Wensveen F Ben-Dor S NKG2D ligands mediate immunosurveillance of senescent cells. Aging (Albany NY) (2016) 8(2):328–44. 10.18632/aging.100897 26878797 PMC4789586

[56] Gur C Doron S Kfir-Erenfeld S Horwitz E Abu-Tair L Safadi R NKp46-mediated killing of human and mouse hepatic stellate cells attenuates liver fibrosis. Gut (2012) 61(6):885–93. 10.1136/gutjnl-2011-301400 22198715

[57] Yoshiji H Kuriyama S Yoshii J Ikenaka Y Noguchi R Nakatani T Tissue inhibitor of metalloproteinases-1 attenuates spontaneous liver fibrosis resolution in the transgenic mouse. Hepatology (2002) 36(4):850–60. 10.1053/jhep.2002.35625 12297832

[58] Bansal R Prakash J De Ruiter M Poelstra K. Interferon gamma peptidomimetic targeted to hepatic stellate cells ameliorates acute and chronic liver fibrosis in vivo. J Control Release (2014) 179:18–24. 10.1016/j.jconrel.2014.01.022 24491909

[59] Bansal R Prakash J Post E Beljaars L Schuppan D Poelstra K. Novel engineered targeted interferon-gamma blocks hepatic fibrogenesis in mice. Hepatology (2011) 54(2):586–96. 10.1002/hep.24395 21538439

[60] Tosello-Trampont AC Krueger P Narayanan S Landes SG Leitinger N Hahn YS. NKp46(+) natural killer cells attenuate metabolism-induced hepatic fibrosis by regulating macrophage activation in mice. Hepatology (2016) 63(3):799–812. 10.1002/hep.28389 26662852 PMC4764418

[61] Radaeva S Sun R Pan HN Hong F Gao B. Interleukin 22 (IL-22) plays a protective role in T cell-mediated murine hepatitis: IL-22 is a survival factor for hepatocytes via STAT3 activation. Hepatology (2004) 39(5):1332–42. 10.1002/hep.20184 15122762

[62] Zenewicz LA Flavell RA Recent advances in IL-22 biology. Int Immunol (2011) 23(3):159–63. 10.1093/intimm/dxr001 21393631

[63] Zenewicz LA Yancopoulos GD Valenzuela DM Murphy AJ Karow M Flavell RA. Interleukin-22 but not interleukin-17 provides protection to hepatocytes during acute liver inflammation. Immunity (2007) 27(4):647–59. 10.1016/j.immuni.2007.07.023 17919941 PMC2149911

[64] Park O Wang H Weng H Feigenbaum L Li H Yin S In vivo consequences of liver-specific interleukin-22 expression in mice: implications for human liver disease progression. Hepatology (2011) 54(1):252–61. 10.1002/hep.24339 21465510 PMC3125432

[65] Zhao J Zhang Z Luan Y Zou Z Sun Y Li Y Pathological functions of interleukin-22 in chronic liver inflammation and fibrosis with hepatitis B virus infection by promoting T helper 17 cell recruitment. Hepatology (2014) 59(4):1331–42. 10.1002/hep.26916 24677193 PMC3970185

[66] Wolk K Kunz S Witte E Friedrich M Asadullah K Sabat R. IL-22 increases the innate immunity of tissues. Immunity (2004) 21(2):241–54. 10.1016/j.immuni.2004.07.007 15308104

[67] Boniface K Bernard FX Garcia M Gurney AL Lecron JC Morel F. IL-22 inhibits epidermal differentiation and induces proinflammatory gene expression and migration of human keratinocytes. J Immunol (2005) 174(6):3695–702. 10.4049/jimmunol.174.6.3695 15749908

[68] Ma HL Liang S Li J Napierata L Brown T Benoit S IL-22 is required for Th17 cell-mediated pathology in a mouse model of psoriasis-like skin inflammation. J Clin Invest (2008) 118(2):597–607. 10.1172/JCI33263 18202747 PMC2200300

[69] Beraza N Malato Y Sander LE Al-Masaoudi M Freimuth J Riethmacher D Hepatocyte-specific NEMO deletion promotes NK/NKT cell- and TRAIL-dependent liver damage. J Exp Med (2009) 206(8):1727–37. 10.1084/jem.20082152 19635861 PMC2722179

[70] Fernandez-Alvarez S Gutierrez-de Juan V Zubiete-Franco I Barbier-Torres L Lahoz A Pares A TRAIL-producing NK cells contribute to liver injury and related fibrogenesis in the context of GNMT deficiency. Lab Invest (2015) 95(2):223–36. 10.1038/labinvest.2014.151 25531568 PMC4310762

[71] Ishiyama K Ohdan H Ohira M Mitsuta H Arihiro K Asahara T. Difference in cytotoxicity against hepatocellular carcinoma between liver and periphery natural killer cells in humans. Hepatology (2006) 43(2):362–72. 10.1002/hep.21035 16440347

[72] Mantovani S Mele D Oliviero B Barbarini G Varchetta S Mondelli MU. NKp30 isoforms in patients with chronic hepatitis C virus infection. Immunology (2015) 146(2):234–42. 10.1111/imm.12495 26094914 PMC4582964

[73] Ochi M Ohdan H Mitsuta H Onoe T Tokita D Hara H Liver NK cells expressing TRAIL are toxic against self hepatocytes in mice. Hepatology (2004) 39(5):1321–31. 10.1002/hep.20204 15122761

[74] Zou Y Bao J Pan X Lu Y Liao S Wang X NKP30-B7-H6 interaction aggravates hepatocyte damage through up-regulation of interleukin-32 expression in hepatitis B virus-related acute-on-chronic liver failure. PLoS One (2015) 10(8):e0134568. 10.1371/journal.pone.0134568 26241657 PMC4524618

[75] Feldstein AE Canbay A Angulo P Taniai M Burgart LJ Lindor KD Hepatocyte apoptosis and fas expression are prominent features of human nonalcoholic steatohepatitis. Gastroenterology (2003) 125(2):437–43. 10.1016/S0016-5085(03)00907-7 12891546

[76] Shimoda S Harada K Niiro H Shirabe K Taketomi A Maehara Y Interaction between toll-like receptors and natural killer cells in the destruction of bile ducts in primary biliary cirrhosis. Hepatology (2011) 53(4):1270–81. 10.1002/hep.24194 21400555 PMC3077894

[77] Tian Z Gershwin ME Zhang C. Regulatory NK cells in autoimmune disease. J Autoimmun (2012) 39(3):206–15. 10.1016/j.jaut.2012.05.006 22704425

[78] Schleinitz N Vely F Harle JR Vivier E. Natural killer cells in human autoimmune diseases. Immunology (2010) 131(4):451–8. 10.1111/j.1365-2567.2010.03360.x 21039469 PMC2999796

[79] Boukouaci W Lauden L Siewiera J Dam N Hocine HR Khaznadar Z Natural killer cell crosstalk with allogeneic human cardiac-derived stem/progenitor cells controls persistence. Cardiovasc Res (2014) 104(2):290–302. 10.1093/cvr/cvu208 25213554

[80] Kumar P Thakar MS Ouyang W Malarkannan S. IL-22 from conventional NK cells is epithelial regenerative and inflammation protective during influenza infection. Mucosal Immunol (2013) 6(1):69–82. 10.1038/mi.2012.49 22739232 PMC3835350

[81] Graubardt N Fahrner R Trochsler M Keogh A Breu K Furer C Promotion of liver regeneration by natural killer cells in a murine model is dependent on extracellular adenosine triphosphate phosphohydrolysis. Hepatology (2013) 57(5):1969–79. 10.1002/hep.26008 22898900

[82] Cosgrove BD Cheng C Pritchard JR Stolz DB Lauffenburger DA Griffith LG. An inducible autocrine cascade regulates rat hepatocyte proliferation and apoptosis responses to tumor necrosis factor-alpha. Hepatology (2008) 48(1):276–88. 10.1002/hep.22335 18536058 PMC4327877

[83] Boulter L Govaere O Bird TG Radulescu S Ramachandran P Pellicoro A Macrophage-derived Wnt opposes Notch signaling to specify hepatic progenitor cell fate in chronic liver disease. Nat Med (2012) 18(4):572–9. 10.1038/nm.2667 22388089 PMC3364717

[84] Bi J Zheng X Chen Y Wei H Sun R Tian Z. TIGIT safeguards liver regeneration through regulating natural killer cell-hepatocyte crosstalk. Hepatology (2014) 60(4):1389–98. 10.1002/hep.27245 24912841

[85] Sun R Gao B. Negative regulation of liver regeneration by innate immunity (natural killer cells/interferon-gamma). Gastroenterology (2004) 127(5):1525–39. 10.1053/j.gastro.2004.08.055 15521020

[86] Almeida CR Caires HR Vasconcelos DP Barbosa MA. NAP-2 secreted by human NK cells can stimulate mesenchymal stem/stromal cell recruitment. Stem Cell Reports (2016) 6(4):466–73. 10.1016/j.stemcr.2016.02.012 27052313 PMC4834048

[87] Cerwenka A Lanier LL. Natural killer cell memory in infection, inflammation and cancer. Nat Rev Immunol (2016) 16(2):112–23. 10.1038/nri.2015.9 26806484

[88] Mandal A Viswanathan C Natural killer cells: in health and disease. Hematol Oncol Stem Cell Ther (2015) 8(2):47–55. 10.1016/j.hemonc.2014.11.006 25571788

[89] Patel P Schutzer SE Pyrsopoulos N. Immunobiology of hepatocarcinogenesis: ways to go or almost there? World J Gastrointest Pathophysiol (2016) 7(3):242–55. 10.4291/wjgp.v7.i3.242 27574562 PMC4981764

[90] Tian Z Chen Y Gao B. Natural killer cells in liver disease. Hepatology (2013) 57(4):1654–62. 10.1002/hep.26115 23111952 PMC3573257

[91] Liu H Deng W Li J Tang Y Zhang L Cui Y Peripheral blood lymphocyte subset levels differ in patients with hepatocellular carcinoma. Oncotarget (2016) 7(47):77558–64. 10.18632/oncotarget.13041 27813499 PMC5363604

[92] Hoechst B Voigtlaender T Ormandy L Gamrekelashvili J Zhao F Wedemeyer H Myeloid derived suppressor cells inhibit natural killer cells in patients with hepatocellular carcinoma via the NKp30 receptor. Hepatology (2009) 50(3):799–807. 10.1002/hep.23054 19551844 PMC6357774

[93] Cai L Zhang Z Zhou L Wang H Fu J Zhang S Functional impairment in circulating and intrahepatic NK cells and relative mechanism in hepatocellular carcinoma patients. Clin Immunol (2008) 129(3):428–37. 10.1016/j.clim.2008.08.012 18824414

[94] Li T Yang Y Hua X Wang G Liu W Jia C Hepatocellular carcinoma-associated fibroblasts trigger NK cell dysfunction via PGE2 and IDO. Cancer Lett (2012) 318(2):154–61. 10.1016/j.canlet.2011.12.020 22182446

[95] Lee SH Miyagi T Biron CA. Keeping NK cells in highly regulated antiviral warfare. Trends Immunol (2007) 28(6):252–9. 10.1016/j.it.2007.04.001 17466596

[96] Su M Huang CX Dai AP. Immune checkpoint inhibitors: therapeutic tools for breast cancer. Asian Pac J Cancer Prev (2016) 17(3):905–10. 10.7314/APJCP.2016.17.3.905 27039716

[97] Hodi FS O’Day SJ McDermott DF Weber RW Sosman JA Haanen JB Improved survival with ipilimumab in patients with metastatic melanoma. N Engl J Med (2010) 363(8):711–23. 10.1056/NEJMoa1003466 20525992 PMC3549297

[98] Pardoll DM. The blockade of immune checkpoints in cancer immunotherapy. Nat Rev Cancer (2012) 12(4):252–64. 10.1038/nrc3239 22437870 PMC4856023

[99] Li H Wu K Tao K Chen L Zheng Q Lu X Tim-3/galectin-9 signaling pathway mediates T-cell dysfunction and predicts poor prognosis in patients with hepatitis B virus-associated hepatocellular carcinoma. Hepatology (2012) 56(4):1342–51. 10.1002/hep.25777 22505239

[100] Zeng Z Shi F Zhou L Zhang MN Chen Y Chang XJ Upregulation of circulating PD-L1/PD-1 is associated with poor post-cryoablation prognosis in patients with HBV-related hepatocellular carcinoma. PLoS One (2011) 6(9):e23621. 10.1371/journal.pone.0023621 21912640 PMC3164659

[101] Wu QW Zhu X Fu X Yang JS Cao ZG Pu C. [Expression of Tim-3 on peripheral CD56(+) NK cells and its correlation with liver fibrosis in patients with advanced schistosomiasis]. Zhongguo Ji Sheng Chong Xue Yu Ji Sheng Chong Bing Za Zhi (2015) 33(5):346–50. 26931038

